# Notes of a protein crystallographer: the advantages of combining new integrated methods of structure solution with traditional data visuals

**DOI:** 10.1107/S205979832101336X

**Published:** 2022-01-24

**Authors:** Celerino Abad-Zapatero

**Affiliations:** a Institute of Tuberculosis Research and Center for Biomolecular Sciences, Department of Pharmaceutical Sciences, College of Pharmacy, University of Illinois at Chicago, Chicago, IL 60607, USA

**Keywords:** teaching, visual aids, pseudo-precession photographs, stereographic projections, crystallography workflows

## Abstract

The suggestion is made that combining analysis using the most advanced crystallographic software with the integrated visual tools of the field will result in more knowledgeable and better trained future generations of structural biologists. The use of integrated visuals could also expedite the structure solution of some recalcitrant and complex macromolecular crystal structures that resist automatic workflows.

## Abstract 2

The streamlined crystallographic software tools for solving macromolecular crystal structures should be complemented with the visual aids that were used in the past (for example precession images and stereographic projections) to facilitate better understanding of the basic crystallographic concepts by younger aspiring crystallographers.

The recent CCP4/APS School in Macromolecular Crystallo­graphy workshop at the Advanced Photon Source (https://www.ccp4.ac.uk/schools/APS-2021/) entitled ‘From data collection to structure refinement and beyond’ brought together in a global electronic meeting some of the most prestigious senior crystallographers, premier method developers and the new generations of aspiring structural biologists and crystallographers. It was a virtual school that also included hands-on data-collection sessions at the Advanced Photon Source and comprised two full weeks of intense immersion in crystallographic methods for the training of younger crystallographers. I decided to register and participate in order to get a refresher course on the newest methods and software tools. I was certainly not disappointed.

Lectures on diffraction theory, the Ewald sphere, data-collection strategies and various methods such as MIR, MR, SIR, SAD *etc.* were presented at a very fast pace. The field is certainly vibrant with computational innovations that will facilitate the solution and refinement of ever more complex structures by conventional crystallography or by the promising youngest branch of the field, cryo-electron microscopy.

However, in my view, the introductory lectures on diffraction theory failed to take advantage of the traditional visual­ization tools that so well illustrate the theory and the basic principles of crystallography such as diffraction theory, the reciprocal lattice and the corresponding symmetry of the different projections. The basic principles were presented (for example the Ewald sphere), but the implications for the visualization of the reciprocal lattice were not adequately illustrated. In particular, the presenters failed to demonstrate the value of the images of the intensity-weighted reciprocal-lattice planes in the different Laue classes. I noticed the absence of highly integrated visual tools along the path to the structure solution of complex structures, including the existence of any noncrystallographic symmetry (NCS) if present (Rossmann & Blow, 1962 ▸). I am referring to precession photos and stereographic projections of the self-rotation function (SRF). Am I a dinosaur? Am I ossified to the point of no redemption? Am I totally out of touch with the new tech­niques and programs that can solve structures automatically using specific and ultrafast pipelines and workflows? Possibly, but I do not think so.

I must add that the writing of this article was not motivated by nostalgia for the ‘good old days’ of tedious data collection and long, interminable and uncertain times for structure solution. My interest is in conveying to software developers, beamline scientists and future generations that the crystallo­graphic community at large will benefit when the superb tools currently available are combined with the more traditional visual guides used in the past.

In this brief essay, I will illustrate this statement with some examples for three reasons. Firstly, although it has been said millions of times and is a mantra in many human endeavors, it is worth mentioning it again: a picture is worth a thousand words. Secondly, because having the younger generations recognize, understand and interpret these images will make them better crystallographers and structural biologists and not just ‘program-running wizards’. Thirdly, but not least, because it will help in solving structures more easily, particularly those complex structures where the automatic workflows cannot find the correct solution for a variety of reasons: low resolution and/or incomplete data, complex NCS, twinning and/or ambiguous space-group assignment, among others.

The precession method was introduced in the 1930s in the laboratory of Professor Martin Buerger at the Massachusetts Institute of Technology. A complete discussion of the method was published by Buerger thirty years later (Buerger, 1964 ▸). It provided a simple and direct way of establishing the space group and unit cell of a crystal by providing undistorted views of the planes of the reciprocal lattice (Fig. 1 ▸ *a*, left and right). The instrument (the precession camera) soon became commercially available and was widely used in crystallography laboratories.

For younger macromolecular crystallographers, it is worth mentioning that the native and heavy-atom derivative data for the determination of the structure of myoglobin at 2 Å resolution were collected by precession photography (Kendrew *et al.*, 1960 ▸). The key advantage was that the successive photographs containing individual planes of the reciprocal lattice comprising the ‘data set’ were very easy to index. The intensities of the corresponding reflections were measured readily using the flatbed densitometers available at the time. The undistorted view of the weighted reciprocal lattice allows immediate indexing as the reflections are arranged regularly along the three main reciprocal axes (Fig. 1 ▸ *a*, left and right). The data for the structure of hemoglobin at 5.5 Å resolution (Perutz *et al.*, 1960 ▸) were collected by precession methods and using an early counter spectrometer (Arndt & Phillips, 1961 ▸), measuring the intensity of one reflection at a time.

Nowadays, you can only see precession cameras in small (museum-like) displays near well established crystallography laboratories: they are a relic of the past. However, current integrated visual software may display the intensity-weighted reciprocal space in almost any possible way with data collected using any hardware or available method.

Younger generations of macromolecular crystallographers are more familiar with the screenless oscillation methods used at synchrotron sources, which were initially used at conventional in-house rotating anodes, collecting the crystallographic reflections from various reciprocal planes simultaneously that could be indexed by the initial software (Arndt & Wonacott, 1977 ▸). The pre-alignment of the crystal axes with respect to the X-ray beam was important to facilitate the indexing and integration (Fig. 1 ▸ *b*). The autoindexing software using Fourier methods that was introduced later (Otwinowski & Minor, 1997 ▸) enormously facilitated the indexing of the initial diffraction patterns and the subsequent data collection and processing for randomly oriented crystals.

Obtaining precession photographs of the main projections of the reciprocal lattice (*i.e. h*0*l*, 0*kl*, *hk*0), often including the upper levels (*i.e. h*1*l*, 1*kl*, *hk*1), was a crucial (and often time-consuming) step in any successful structure determination. This was possible by the careful analysis and visual inspection of these images (Figs. 2 ▸ *a*–2 ▸ *c*, 3 ▸ *a* and 3 ▸ *b*). I dare say that the majority of younger crystallographers will not even know what I am talking about. Yet all of these data, planes and projections are ‘buried’ in the well known ‘mtz’ files produced by the processing programs, namely *MOSFLM*/*iMOSFLM* (Winn *et al.*, 2011 ▸), *HKL*-2000/*HKL*-3000 (Otwinowski & Minor, 1997 ▸) and *XDS* (Kabsch, 2010 ▸). They can be examined using the *VIEWHKL* program available in various software packages (*CCP*4 suite; Winn *et al.*, 2011 ▸), as well as the abovementioned *HKL*-3000 (Minor *et al.*, 2006 ▸) and *Phenix* suite (Liebschner *et al.*, 2019 ▸). The precession photographs allow the immediate calculation of the unit-cell parameters, and by looking at the pattern of intensities of certain sets of reflections (for example axial reflections: 00*h*, 0*kl*, 00*l*) the presence of screw axes of symmetry can be determined, as well as any lattice centering by examining the systematic absences (Figs. 2 ▸ *a*–2 ▸ *c*, 3 ▸ *a* and 3 ▸ *b*).

This is what the current crystallographic programs do; it is not magic. In addition, the programs compare the intensities of what they think are symmetry-equivalent reflections and provide reasonable suggestions for the full symmetry of the crystal lattice based on some statistical inferences. All of this is important and valuable but, in my experience, it always helps to look at these ‘pseudo’ precession photographs to confirm what the programs are suggesting (Figs. 2 ▸ *a*–2 ▸ *c*, 3 ▸ *a* and 3 ▸ *b*). Except for enantiomorphic space groups (for example *P*6_1_ versus *P*6_5_), this is how the software proposes a space group and will merge the integrated reflections if the statistical parameters are robust enough.

Nowadays, view-enabling programs such as *HKL*-3000 and *Phenix* can also be used to display various views, regions and planes of the reciprocal lattice (2D or 3D viewers in glowing colors!), including the completeness of the reciprocal data collected in three dimensions. This is very useful to identify certain regions of reciprocal lattice where significant ‘gaps’ exist due to the crystal orientation during data collection. These gaps or wedges can lead to incorrect space-group determination, particularly if the missing data are along the axes. This is something that the old precession photographs would not allow us to do (Fig. 2 ▸ *c*).

The article by Rossmann and Blow defining the ‘self-rotation function’ (SRF) and its usage for ‘the detection of sub-units within the crystallographic asymmetric unit’ is a classic (Rossmann & Blow, 1962 ▸). The final sentence of the abstract establishes what was to become the most common application of this concept: ‘application of the *R* function to horse haemoglobin gives a dominant peak that corresponds accurately to the relative orientation of the α and β chains’. Attempts to solve other protein structures in the years that followed often encountered the problem of having more than one molecule in the asymmetric unit with or without any point-group symmetry. Solving this problem was a key step in unraveling the heavy-atom sites and subsequent phase calculations and refinement. One of the most relevant examples of these years was the use of the rotation function for the discovery of a noncrystallographic twofold axis of symmetry in rhombohedral insulin (Dodson *et al.*, 1966 ▸). This illustration demonstrated the value of calculating and interpreting the SRF in the early stages of structure determination. It was used routinely in the 1970s and beyond to establish the orientation of the symmetry elements in icosahedral viruses (point group 532), where it was crucial for the averaging of the initial electron-density maps and phase improvement. A time perspective on the molecular-replacement method and its applications has recently been published (Dodson, 2021 ▸).

Certainly, the new software packages do calculate the SRF to consider the presence of NCS and they routinely list the various NCS axes, typically using Eulerian angles, but the relative relationship of the NCS peaks is difficult to visualize using the Euler angles (α, β, γ or θ1, θ2, θ3). It is certainly easier using the polar coordinates of the axial directions (φ, ψ and the angle of rotation κ or χ). I routinely use the option in *MOLREP* (Vagin & Teplyakov, 1997 ▸, 2010 ▸; Winn *et al.*, 2011 ▸) to calculate the SRF and plot it on a stereographic projection (Wulf net), which provides a superb overview of the crystallographic and NCS elements and their relative orientations with respect to the crystallographic axes. In my experience, these stereographic projections are very valuable to (i) characterize the NCS elements and their relative orientations with respect to the crystallographic axes (Fig. 4 ▸); (ii) discern issues with the quality of the crystallo­graphic data, such as the degree of twinning (Fig. 5 ▸); (iii) identify the correct symmetry of the crystal lattice (Fig. 6 ▸) and (iv) unravel complex NCS symmetry in low-symmetry space groups, including the presence of pseudo-symmetry, to facilitate structure solution (Fig. 7 ▸). I will illustrate these four cases with examples.

The orientation of the NCS symmetry elements in the lattice is the most traditional use, and in simple cases it is very easy to interpret using the direct listing from the programs. However, even the common presence of a 222 tetramer in the asymmetric unit can be challenging when none of the three twofold axes are aligned with the crystallographic symmetry. Fig. 4 ▸ illustrates the SRF of a crystal of the class II fructose 1,6-bisphosphatase (FBPase) from the pathogenic bacterium *Francisella tularensis*. The protein is present in solution as a tetramer of identical sub­units and quite often the entire tetramer is present in the asymmetric unit of crystals belonging to space group *P*1. In the illustration, there are actually two tetramers with 222 symmetry within the unit cell in slightly different orientations and separated by about 70 Å along the *c* axis.

I have often found that obtaining the SRF immediately after the crystallographic data have been reduced helps to recognize ‘red flags’ regarding the quality of the data; this can be a significant time saver for a project. A lack of distinct features and the absence of the symmetry elements expected from the data reduction is often a reflection of disordered crystals and/or certain types of twinning. In certain cases, it is possible to select the ‘best crystal’ data set to proceed based on inspection of the SRF. Fig. 5 ▸ illustrates how different degrees of twinning in two different data sets of *P*2_1_ crystals grown in the presence of Mg^2+^ (not the native divalent cation) are reflected in the appearance and interpretation of the SRF.

The most unexpected and revealing usage of the SRF has been to show the symmetry elements of the crystallographic data without any preconceived idea of the lattice. Of course, the auto-indexing programs provide reasonable guesses of the crystal lattice based on the values of the unit-cell parameters (dimensions and angles) and these are critical constraints. However, I encountered an example where the auto-indexing and subsequent refinement indicated a tetragonal *P*4_1_22 (or enantiomer) lattice and yet I could not solve the structure, even though it was a rather simple MR solution of a protein N-terminal domain of about 150 amino acids. A rather straightforward calculation *and* plotting the SRF immediately showed an unambiguous threefold axis of symmetry along the approximate body diagonal of the cell, directly pointing to a cubic lattice (Fig. 6 ▸). The structure was solved immediately after reprocessing the data in *P*4_1_32. In cases of space-group ambiguity, I suggest that processing the initial crystallo­graphic data in lower symmetry space groups, within the same or different Laue classes, and examination of the SRF could point to the correct space-group assignment, in conjunction with the suggested solutions provided by the auto-indexing programs.

The dramatic impact of the use of the SRF to unravel the NCS of complex asymmetric units is presented in Fig. 7 ▸. The crystallographic data could be reduced either in *P*2_1_ (unit-cell parameters *a* = 80.1, *b* = 130.3, *c* = 112.6 Å, β  = 90.07°) or in *P*222_1_, with the longest *c* axis coinciding with the 2_1_ screw axis. The NCS revealed significant peaks for twofold, threefold and even sixfold symmetries inside the asymmetric unit. The solution by MR proceeded in a stepwise fashion, as reported previously (Wolf *et al.*, 2020 ▸; PDB entry 6pbs). It was particularly intriguing to see strong evidence for a threefold axis, as only dimers had been hinted at before. Finally, the solution was obtained and refined by solving for a 32 symmetrical cluster (six chains) in *P*222_1_ indexing and searching for two such clusters in the diffraction data reduced as *P*2_1_ (*b* unique). The biological surprise appeared when each poly­peptide chain was bound to two ‘ligand’ molecules, making an asymmetric unit of 12 (ClpC1 NTD chains) and 24 (ecumicin) molecules (1:2 stoichiometry; Wolf *et al.*, 2020 ▸).

Concurrent with the previous examples, it is worth presenting the following argument. The issues related to how to teach crystallography have been brought up for discussion many times. There are fewer formal courses of crystallography in the curricula that are important for training future students, even in the more traditional areas of biophysics, biophysical and analytical methods of structure determination, physical chemistry and others. The most common training occurs when graduate students encounter a need to solve a biological problem related to a macromolecule (protein/nucleic acid) of biological interest. The common theme is that solving the structure of the target molecule will provide insights into its function, whether catalytic, regulatory or other. Thus, the student is asked to attempt to clone, express, purify, crystallize and solve the structure of the macromolecule of interest. Often, there is no expertise in the field in the laboratory, and once the students manage to obtain crystals they are supposed to solve the structure with the ‘powerful software’ tools available. The assumption is that once the data have been collected and processed (possibly even by beamline scientists), the structure solution comes from running a series of programs. The focus is on solving the structure, and ‘learning’ crystallography is often equated to being able to run a series of programs, often as streamlined pipelines, and ending with the structure ready for ‘interpretation’ and rationalizing the biological function(s). Unfortunately, this approach does not teach crystallography, and this is the reason why workshops and crystallography schools are so useful. However, in addition to what those schools do very well, it is suggested that they also include use of the very valuable visual tools of the past to illustrate and explain more fully the crystallographic concepts underlying the amazing software tools that they are providing to younger generations. This suggestion should also be adopted by the courteous beamline scientists who often process the collected data for inexperienced users.

In summary, images are more valuable than words and various lists of copious numbers. They are invaluable as a complement to the extensive computer outputs. Certain ‘traditional’ crystallographic images are more informative than the various Cartesian plots produced by most crystallographic programs. I would ask software developers that in addition to the extensive output of their workflows, additional graphic files are output to allow the users the option to examine the three main projections of the reciprocal lattice and an image of the stereographic projection of the SRF using the integrated intensities. I would also encourage the beamline personnel and younger generations to use the integrated visualization tools available to ‘cross-examine’ the answers inferred by the programs. This visual information would perfectly complement the numerical and statistical output of these programs and would probably aid in space-group determination and in the better characterization of noncrystallographic symmetry, if present. Together, these additions will be a more reliable and informative path to the satisfying experience of solving the structure, with a complete understanding of the process and a fuller appreciation of the final result.

## Figures and Tables

**Figure 1:**
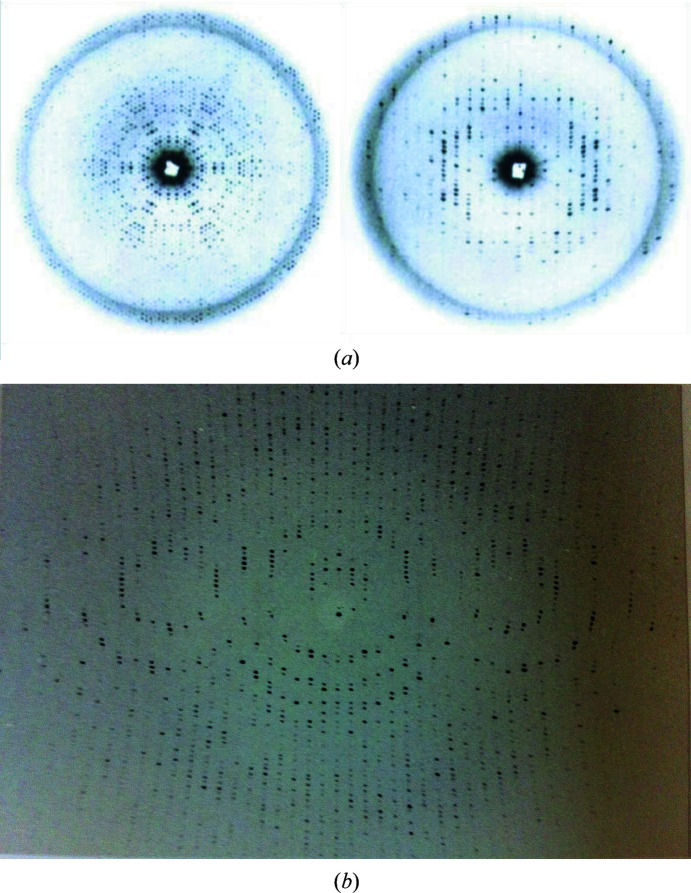
(*a*) Precession photographs of the two main planes of the reciprocal lattice (left, *hk*0; right, *h*0*l*) of crystals of C-phycocyanin, a blue-colored protein extracted from the green alga *Agmenellum quadruplicatum*. The precession angle is μ = 9°. These diffraction patterns were obtained with an overnight exposure on a precession camera model from the 1970s manufactured by the Charles Supper Co., Boston, Massachusetts, USA mounted on an Elliot rotating-anode generator. Note the 6*mm* symmetry of the diffraction pattern along the unique *c* trigonal axis (left) and the centrosymmetric pattern for the *h*0*l* plane (right), with the X-rays perpendicular to the main symmetry axis (threefold). A very small beam stop was used to visualize the lowest resolution reflections to understand the packing of the 32 molecular aggregates (Hackert *et al.*, 1977 ▸). In the *h*0*l* reciprocal-lattice plane (right) the main axis (00*l* reflections) is horizontal. (*b*) Oscillation diffraction pattern (Δφ = 1.5–3.5°) of the same crystal form as in (*a*), with the *c* axis of the crystal oriented perpendicular to the X-ray beam (crystal-to-film distance 7.5 cm). The central lune corresponds to the *h*0*l* plane shown on the right in (*a*). The crystallo­graphic parameters of the unit cell were *a* = *b* = 184.5, *c* = 60.5 Å (trigonal space group *P3*21).

**Figure 2:**
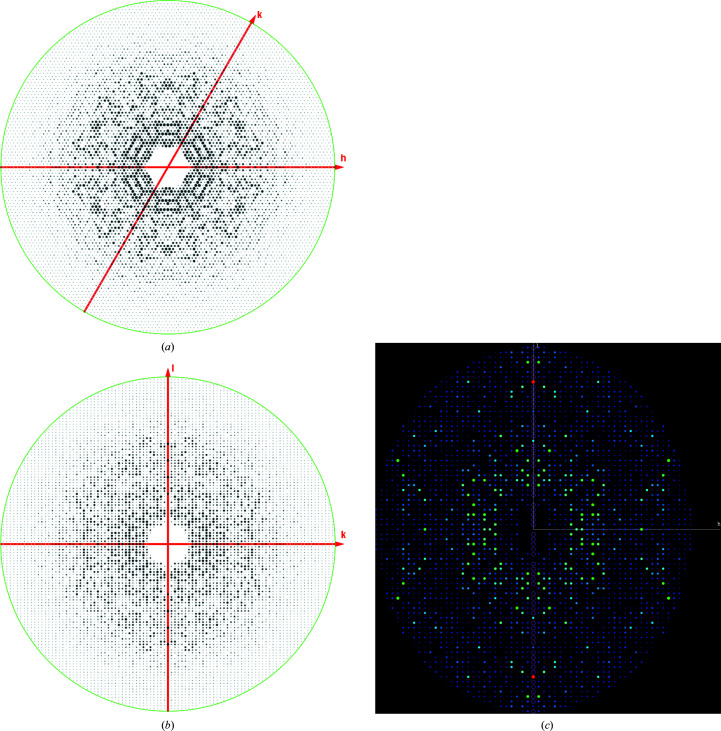
Pseudo-precession photographs extracted using *VIEWHKL* from the processed data of *Mycobacterium tuberculosis* FBPase crystals (space group *P*6_1_22, unit-cell parameters *a* = *b* = 129.8, *c* = 141.2 Å). (*a*) *hk*0 plane exhibiting 6*mm* symmetry. (*b*) 0*kl* plane with the 00*l* reflections vertical; notice the systematic extinctions 00*l* = 6*n* demonstrating the presence of the *P*6_1_ (or *P*6_5_) screw axis (Wolf *et al.*, 2018 ▸). (*c*) *h*0*l* plane (00*l* reflections vertical) as rendered by the software available in *Phenix* (rainbow mode) showing the systematic absences (empty circles) along the main screw axis (different crystal but same space group). Reflection intensity ranges from blue to red (weak to strong).

**Figure 3:**
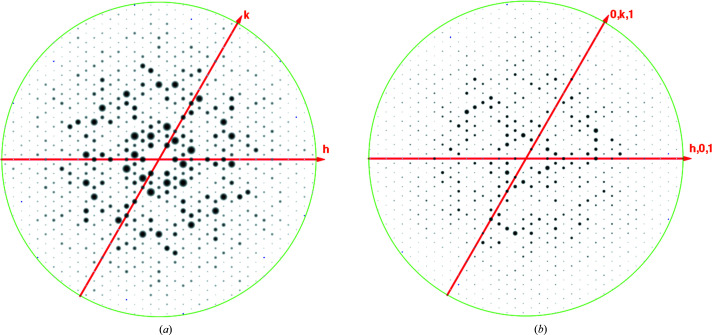
Pseudo-precession photographs produced by *VIEWHKL* of data collected at the APS (LS-CAT) for the protein holo-(acyl-carrier-protein) synthetase (ACPS) from *M. smegmatis.* The program uses the reduced data in the ‘mtz’ file and extracts the corresponding reflections to display specific planes of the reciprocal lattices, such as *hk*0, *hk*1, *hhl* *etc.*, creating ‘pseudo-precession’ photographs. The data were integrated and processed using the beamline software (*HKL*-2000). The space group was confirmed to be *R*3. These two pseudo-precession photographs illustrate the power of the precession images to identify the space group in a dramatic manner. (*a*) The *hk*0 view of the weighted reciprocal lattice along the main threefold axis showing sixfold symmetry. (*b*) In contrast, the ‘upper level’ photograph *hk*1 only displays threefold symmetry. This is diagnostic of trigonal space groups. Other crystallographic suites of programs such as *HKL*-3000 and *Phenix* (see text for details) can be used to effectively display the processed crystallographic data to complement the printed output of the crystallo­graphic workflows.

**Figure 4:**
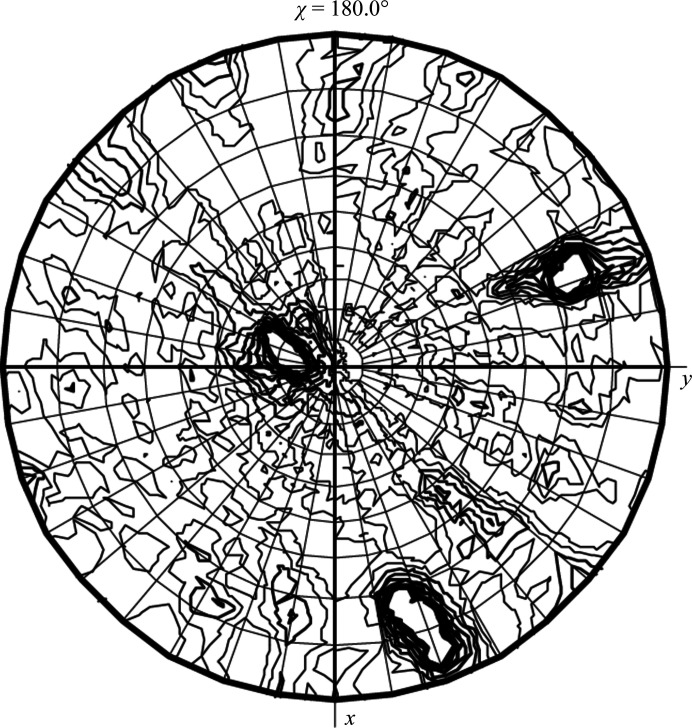
NCS present in the *P*1 unit cell of Mn^2+^-containing crystals of *F. tularensis* FBPase. The stereographic projection (χ = 180°) shows the presence of three broad, nearly orthogonal twofold peaks in a general orientation with respect to the crystal axes (*a* = 65.82, *b* = 76.37, *c* = 141.11 Å, α = 76.97, β = 87.10, γ = 75.78°). The solution of the structure confirmed the presence of two 222 tetramers with very similar orientations in the *P*1 cell, as indicated by the peak broadening (Selezneva *et al.*, in preparation).

**Figure 5:**
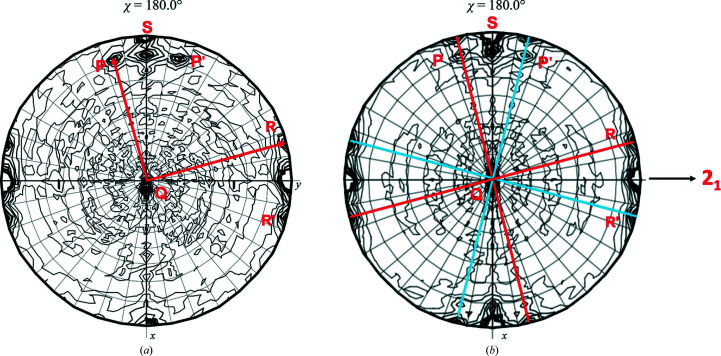
Degree of twinning and orientation of the tetramer in the *P*2_1_ unit cell of Mg^2+^-containing crystals of *F. tularensis* FBPase. (*a*) SRF of data collected from crystal *A*, for which the twinning law was impossible to estimate. (*b*) SRF from crystal *B* that was interpreted as two 222 tetramers (axes directions labeled as *PQR* and *P*′*Q*′*R*′ related by a crystallographic 2_1_ screw axis; horizontal) with a twinning fraction of 0.433 as refined by *REFMAC*5 (Murshudov *et al.*, 2011 ▸). The structure was solved and fully refined (PDB entry 7js3). Reproduced with permission from Selezneva *et al.* (2020 ▸).

**Figure 6:**
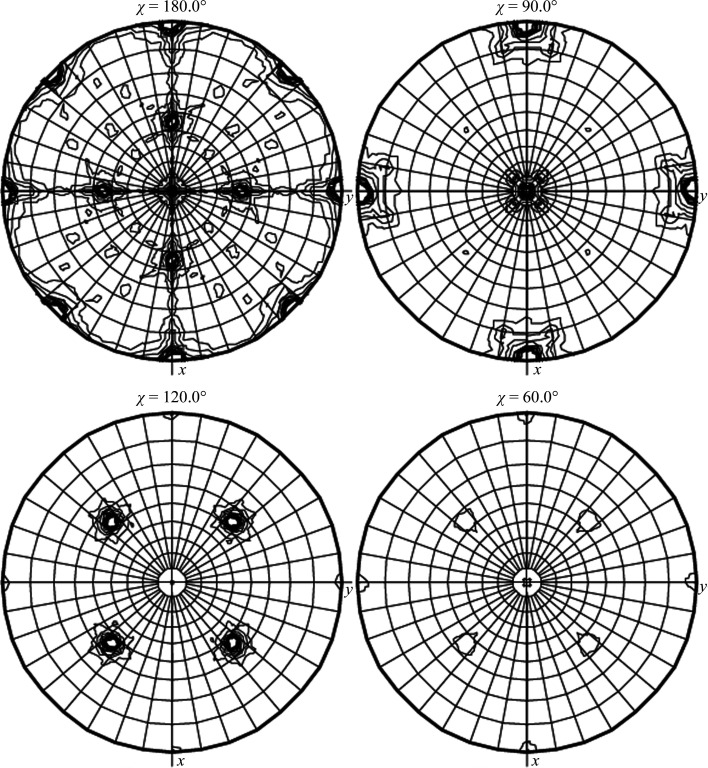
SRF of the crystallographic data for ClpC1 NTD–rufomycin processed in space group *P*4_1_22, showing the threefold approximately along the body diagonal of the crystallographic unit cell (bottom left) as well as the inclined twofold axis (top left). Notice that since the crystallographic axes are not exactly equal (in this tetragonal system) the orientation of the ‘crystallographic’ threefold axis is not exactly at 45° from each of the axes *x*, *y* and *z*. It would have been oriented exactly at the body diagonal if the data had been processed in *P*4_1_32 (the correct space group). It should be emphasized that the initial data integration and processing was performed in the tetragonal space group with acceptable *R*_merge_ values in all resolution ranges. The crystals appeared to be tetragonal with no obvious threefold symmetry. Only after the failure to solve the structure was the space-group symmetry questioned. The *R*_merge_ values from the cubic processing were not significantly better, although the redundancy increased.

**Figure 7:**
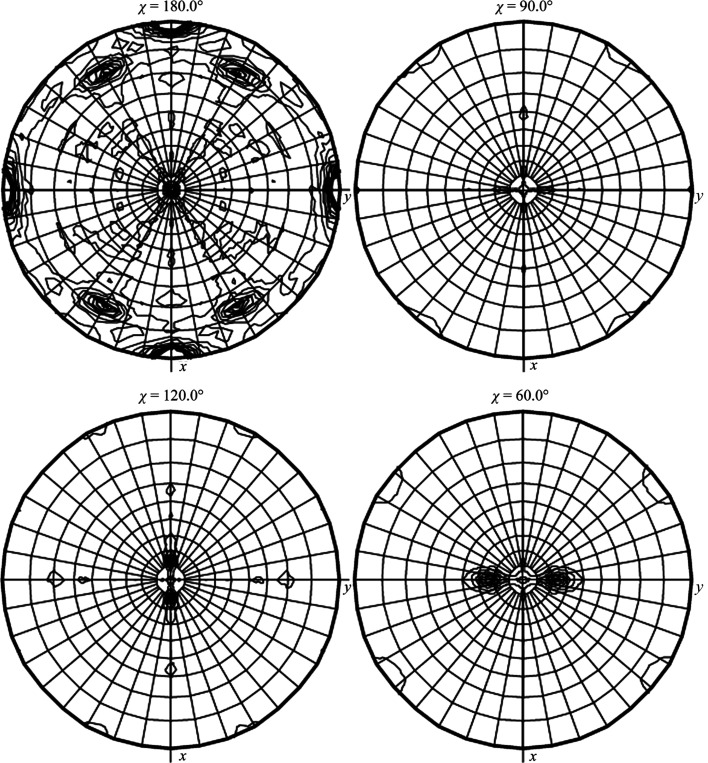
The complex NCS present in the crystals of ClpC1 NTD–ecumicin. The asymmetric unit of the crystal of ClpC1 NTD–ecumicin (space group *P*2_1_) contained 12 molecules of the NTD domain of ClpC1 and 24 molecules of the ligand, the natural depsipeptide ecumicin. The 12 ClpC1NTD–ecumicin (1:2) complexes were packed in a nearly orthorhombic cell in two clusters of approximately 32 aggregates (Wolf *et al.*, 2020 ▸).
